# Reducing perioperative red blood cell transfusion in adult aortic surgery: innovative application and process optimization of autologous plateletpheresis

**DOI:** 10.1007/s44254-025-00126-1

**Published:** 2025-09-13

**Authors:** Jie Gao, Xurong Gao, Cuntao Yu, Hongwen Ji

**Affiliations:** 1 Department of Anesthesiology, Fuwai Hospital, Chinese Academy of Medical Science and Peking Union Medical College, Beijing, 100037 China; 2 Department of Transfusion Medicine, Fuwai Hospital, Chinese Academy of Medical Science and Peking Union Medical College, Beijing, 100037 China; 3 Department of Cardiovascular Surgery, Fuwai Hospital, Chinese Academy of Medical Science and Peking Union Medical College, Beijing, 100037 China

**Keywords:** Autologous plateletpheresis, Patient blood management, Blood transfusion

## Abstract

**Purpose:**

Coagulopathy is a common perioperative complication in aortic surgery, increasing the risk of bleeding and transfusion requirements. This study aimed to evaluate the impact of autologous plateletpheresis on reducing perioperative red blood cell (RBC) transfusion rates in adult aortic surgery patients.

**Methods:**

This prospective, single-center, single-blind randomized controlled trial enrolled 134 participants undergoing aortic surgery with cardiopulmonary bypass, randomized in a 1:1 ratio. The primary outcome was the perioperative RBC transfusion rate and covariates included patient preoperative characteristics and intraoperative factors. Multivariable logistic regression models of the relative risk were evaluated.

**Results:**

The intervention group demonstrated several clinical advantages, including significantly reduced perioperative blood transfusion requirements, lower Factor VII usage, and shorter surgical duration (all *p* < 0.05). Storage of autologous platelet in citrate-containing bags resulted in increased calcium administration (median 3.00g vs 2.00g; *p* < 0.05) and prolonged time between central venous catheter placement and heparinization in aortic root surgery (52.14 ± 7.75 vs 42.15 ± 6.13 min; *p* < 0.001).

**Conclusion:**

The autologous plateletpheresis technique reduces transfusion requirements, shortens surgical duration, enhances clinical outcomes, and accelerates recovery. However, careful calcium ion monitoring and coordination of pre-CPB preparation times are essential to maintain surgical workflow.

**Trial Registration:**

Registered at the Chinese Clinical Trial Registry on November 16, 2022 (ID ChiCTR2200065834, https://www.chictr.org.cn/showproj.html?proj=185761).

**Supplementary Information:**

The online version contains supplementary material available at 10.1007/s44254-025-00126-1.

## Introduction

Perioperative coagulopathy and hemorrhage present significant challenges in cardiovascular surgery, particularly in complex aortic surgeries. Epidemiological data from the United States indicate a significant increase in transfusion rates among cardiac surgery patients, rising from 12.3% in 1999 to 34% by 2010, with aortic procedures constituting the majority of cases [1]. In 2019, a total of 326 aortic surgeries were performed in our center, with 236 (72.39%) cases receiving transfusion. These challenges stem from a multitude of factors, including coagulation factor depletion, systemic inflammation, activation of fibrinolysis, ischemia-reperfusion injury, surgical trauma, deep hypothermic circulatory arrest, and prolonged cardiopulmonary bypass (CPB) [2, 3], together contribute to the development of acquired thrombocytopenia and platelet dysfunction [4].

Platelets, fundamental in maintaining vascular integrity, preventing spontaneous bleeding, and mediating primary hemostasis through glycoprotein receptors, have been extensively studied [5, 6]. Given the critical role of platelets in hemostasis, platelet infusion remains a cornerstone therapy for managing thrombocytopenia or platelet dysfunction in patients requiring surgical intervention [7].

The use of allogeneic transfusion, while potentially life-saving, carries dose-dependent risks. These include pulmonary complications, infections, transfusion-related circulatory overload, prolonged mechanical ventilation, extended hospital stays, increased overall hospitalization costs, and in-hospital mortality among patients undergoing cardiovascular surgery [8]. As a result, the adoption of Patient Blood Management (PBM) program emerges as a viable strategy to preserve platelet resources in these patient populations [9].

Autologous platelet-rich plasmapheresis (aPRP) stands out as a novel approach to autologous blood transfusion. According to the 2011 recommendations of the Society of Thoracic Surgeons and the Society of Cardiovascular Anesthesiologists, aPRP could be considered a reasonable strategy for blood conservation, given the provision of a dependable blood supply (class IIa, level of evidence A) [10].

However, debates have arisen regarding its effectiveness and applicability. The process necessitates a substantial amount of blood, approximately 20–30% of the total blood volume, for adequate platelet collection. This requirement may pose challenges such as circulatory instability and exacerbation of blood dilution due to fluid overload. In contrast, autologous platelet concentrate (APC) offers higher platelet counts compared to aPRP and imposes minimal impact on hemodynamics during the harvesting process. Our study hypothesizes that implementing standardized protocols for APC collection can effectively reduce perioperative allogeneic transfusion requirements in patients undergoing aortic surgery while minimizing the risk of perioperative adverse events.

## Methods

### Study design and patients

This prospective, single-center, single-blind randomized controlled trial was approved by the Fuwai Hospital's Institutional Review Board (approval NO. 2022–1806) and written informed consent was obtained from all subjects participating in the trial. The trial was registered prior to patient enrollment at chictr.org.cn (ChiCTR2200065834).

Inclusion criteria for eligible participants were as follows: (1) scheduling for elective aortic arch surgery, (2) American Society of Anesthesiologists classification I–III, (3) age between 18 and 65 years, body weight more than 50 kg, (4) platelet counts between 150–450 × 10^9^/L, and (5) a willingness to provide informed consent for participation in the study. Patients would not be eligible for enrollment if they presented with any of the following conditions: (1) a history of platelet donation within 15 days before surgery, (2) preoperative cardiogenic shock, cardiac arrest, severe systolic hypotension, an oxygen saturation of mixed venous blood below 75%, or dependence on mechanical circulatory support, (3) thrombocytopenia, platelet dysfunction, or any known history of a bleeding disorder, (4) thromboembolic diseases, (5) intellectual or legal disabilities, (6) severe renal impairment (serum creatinine level > 3.3 mg/dL), (7) stroke, (8) vitamin K and/or vitamin C deficiency, (9) known allergies or contraindications to citrate anticoagulants or their components, (10) trauma with multiple organ injury, or (11) concurrent enrollment in another perioperative interventional study.

All participants were allocated into two groups: the intervention group (also referred to as the APC group, defined as those who underwent preoperative autologous plateletpheresis) and the control group (defined as the blank control group, with no intervention), in a 1:1 ratio using a simple randomization process. All procedures performed in the study complied with the Declaration of Helsinki and adhered to the Consolidated Standards of Reporting Trials (CONSORT) guidelines.

### Sample size calculation, randomization, blinding and data collection

Based on the analysis of data from adult aortic surgeries conducted at our center in 2019, a total of 326 aortic surgeries were performed, with 236 cases receiving packed red blood cell (pRBC) transfusion. A conservative estimate has been made to ensure adequate statistical power, that the perioperative pRBC transfusion rate in the APC group could be reduced by 25% compared to the control group, resulting in a decrease from 72.39% to 47.39%. A sample size of 120 patients was calculated, considering a significance level of α = 0.05, a power of 0.80, and randomization in a 1:1 ratio. To account for possible crossovers, protocol deviations, and an estimated 10% dropout rate, the expected enrollment for the clinical trial is 134 patients.

A simple randomization method was used to assign all participants in a 1:1 ratio to either the APC group or the control group. Participants included in the formal study were randomly assigned using computer-generated randomization software, and the results were placed in opaque envelopes. Each envelope was labeled with a two-digit random number. Following the order of participant enrollment, the envelopes were opened sequentially according to the ascending order of the random numbers on the envelope covers, thereby determining the participant’s group allocation.

The methodological constraints of this study need collaboration with anesthesiologists and precluded the feasibility of double-blinding due to the interventional nature. Also, the technical requirements excluded the possibility of implementing a placebo control group. So we adopted a single-blind design, maintaining blinding for surgical teams, intensive care unit (ICU) physicians, nursing staff, data collectors, and statistical analysts throughout the trial period.

Data collection occurred at various time points: after central venous catheterization (T0), before heparinization (T1), at the end of surgery (T2), 24 h post-surgery (T3), 48 h post-surgery (T4), and 72 h post-surgery (T5), extending until the patient's discharge. At T0–T5, 8 mL of venous blood was collected and corresponding laboratory results were recorded.

### Anesthesia induction and maintenance

All eligible participants received a standardized anesthesia method with continuous vital sign monitoring, including electrocardiogram, oxygen saturation, and invasive blood pressure obtained from the left radial artery/brachial artery and left dorsal pedis artery/femoral artery upon entering the operating room. Baseline hemodynamic parameters were measured and recorded, including bispectral index (BIS) and regional cerebral oxygen saturation. Intubation was induced using midazolam (0.05–0.1 mg/kg), etomidate (0.2–0.3 mg/kg), sufentanil (0.5–1 μg/kg), and cisatracurium (0.2 mg/kg). We implemented a protective ventilation strategy that involved maintaining a tidal volume of 6–7 ml/kg, adjusting positive end-expiratory pressure to 4–8 mmHg, setting the fraction of inspired oxygen at 0.5–1.0, and regulating the ventilation rate to keep the end-tidal partial pressure of carbon dioxide within the range of 35–45 mmHg. Body temperature was monitored using both nasal and rectal probes. The dosages of propofol, dexmedetomidine, and sevoflurane were set to sustain a BIS between 40 and 60. Additionally, intermittent doses of sufentanil (0.5–1.0 μg/kg) and cisatracurium (50 μg/kg) were administered as needed. After intubation, both groups received an 8.5 Fr three-lumen central venous catheter and an 8.5 Fr Swan-Ganz catheter via the right internal jugular vein. The process of blood transfusion was diligently overseen, adhering to the center's established protocols. Standardized intraoperative blood conservation techniques were applied in both groups. Lactated Ringer’s solution was used intraoperatively to maintain intravascular volume and hemodynamic stability. Hematocrit was maintained at 20–24% during CPB, 25–28% before weaning from CPB, and generally above 27% in the ICU, adjusted based on clinical status.

### Interventions

**Fig. 1:**
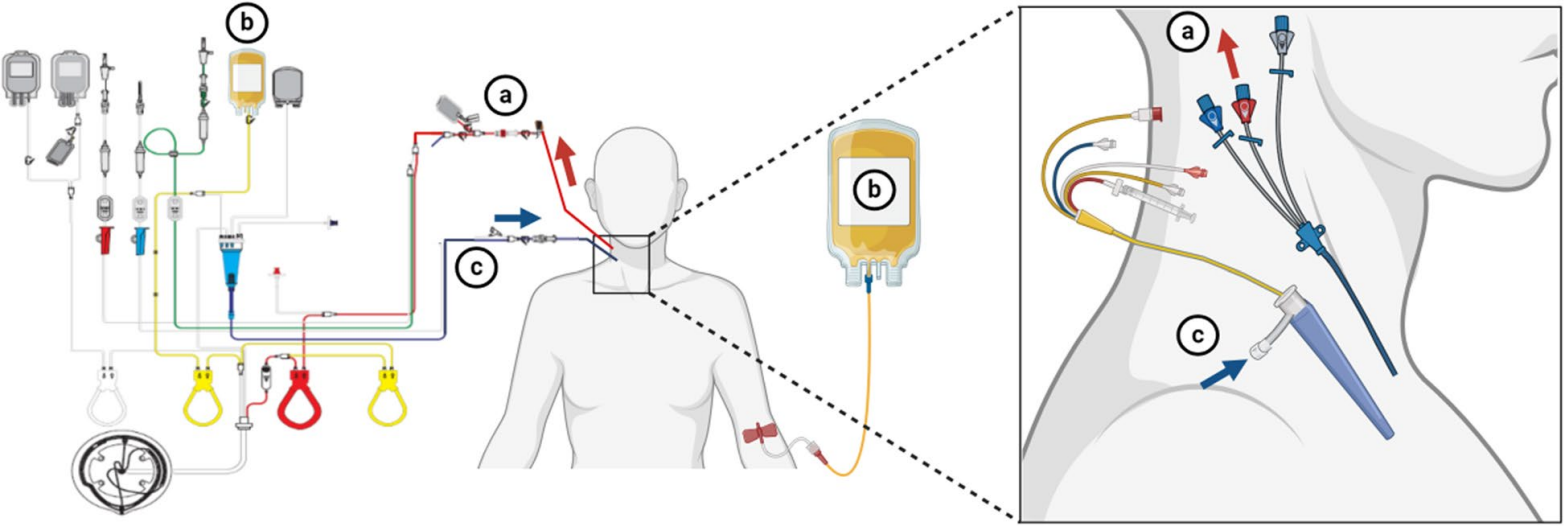
Diagram of centrifugation and autologous platelet collection. **a** The distal end of central venous catheter is connected to the blood cell separator tubing, with the blood flow direction from the patient to the blood cell separator. **b** The collected autologous platelets are transferred into a storage bag. **c** The Swan-Ganz catheter infusion port is connected to the blood cell separator tubing, with the blood flow direction from the blood cell separator to the patient. Note: The Swan-Ganz catheter is placed according to the patient's needs, and the infusion port must be positioned. The diagram is for illustrative purposes only

**Fig. 2:**
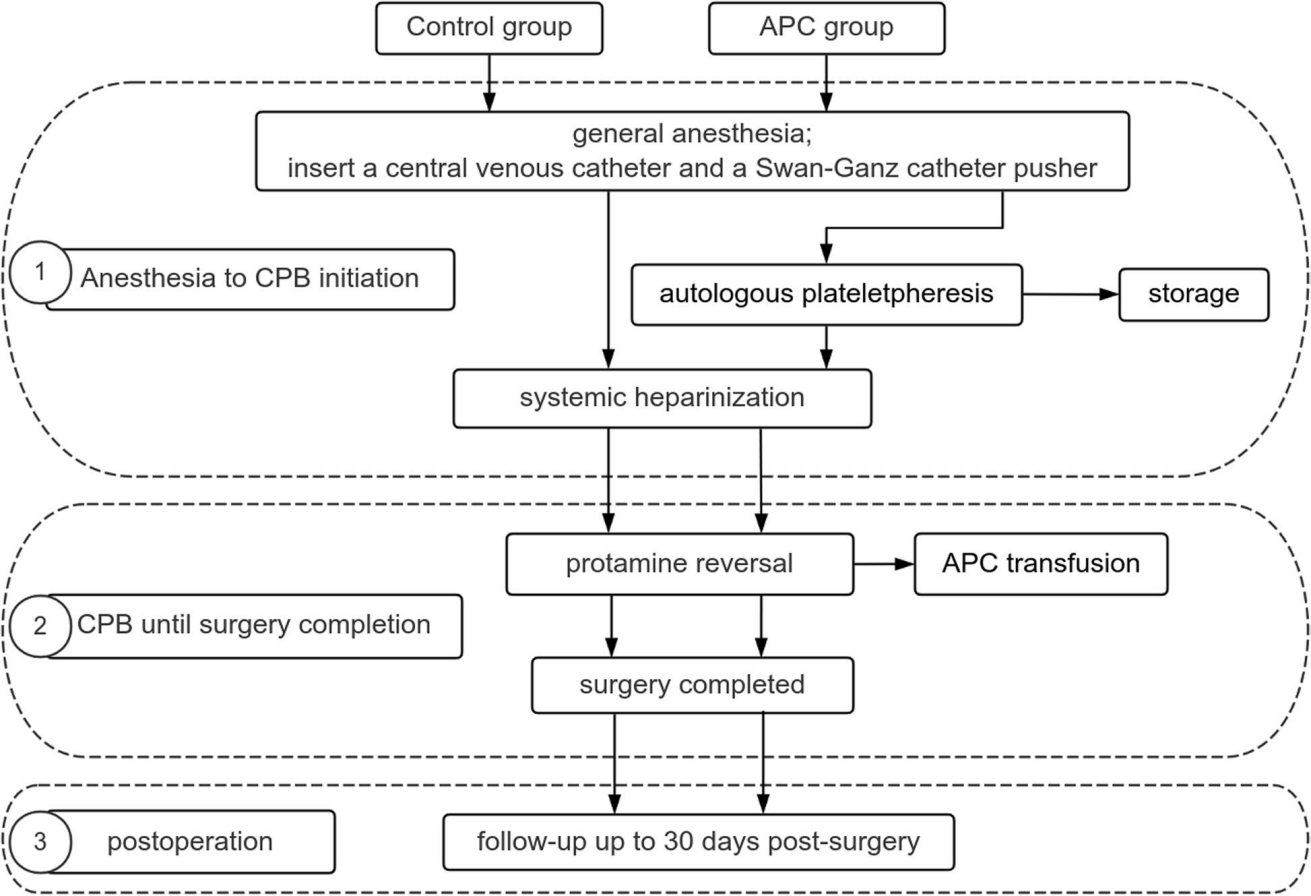
The methodology flow chart of the study. *APC* autologous platelet concentrate, *CPB* cardiopulmonary bypass

**Fig. 3:**
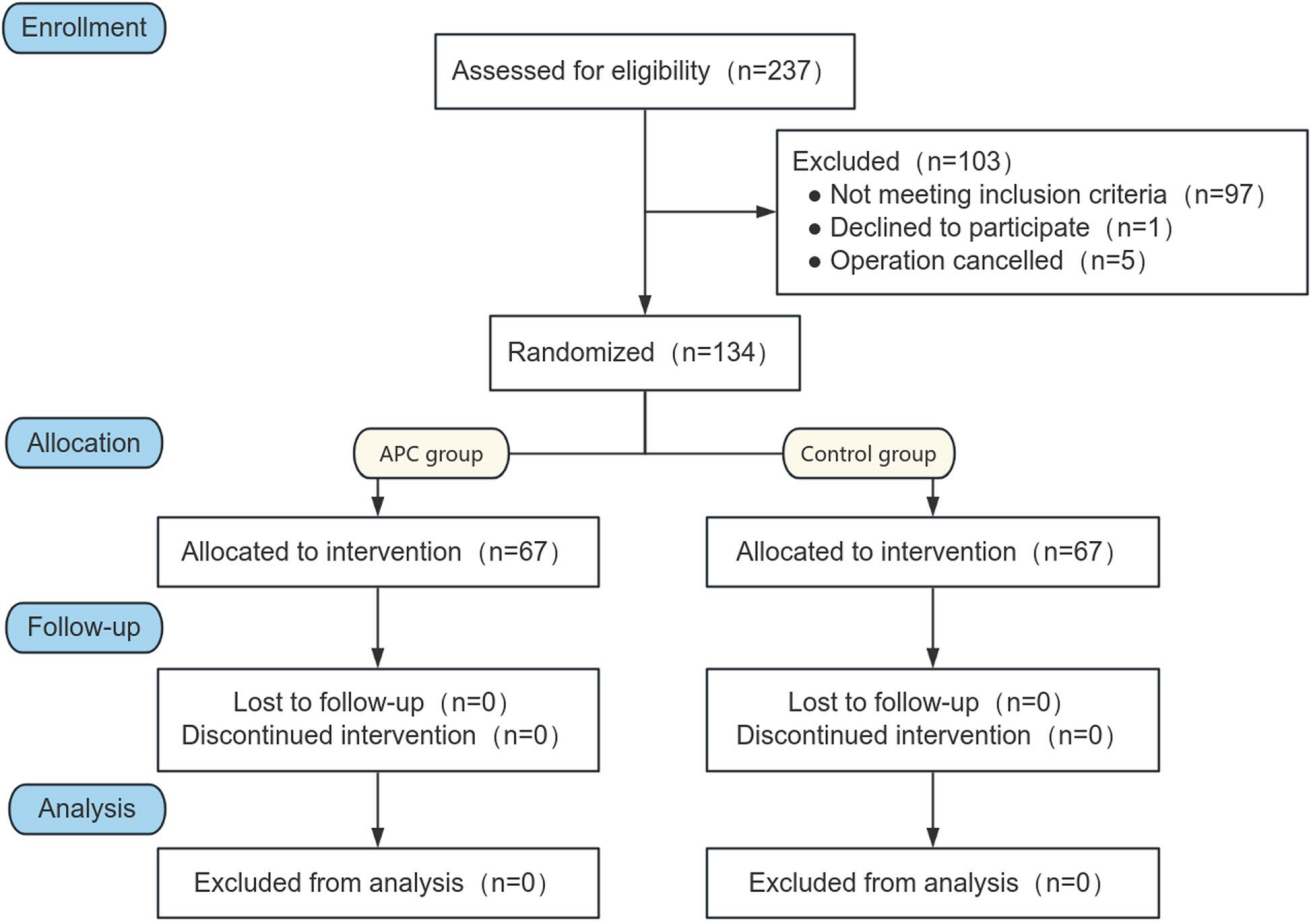
The CONSORT flow chart of the study. *APC* autologous platelet concentrate

Following the induction of general anesthesia, a platelet separation device (Fresenius Kabi, COM.TEC, equipped with disposable separator pipeline consumables of Fresenius C5L) was connected to the central vein and Swan-Ganz catheter. The blood collection rate, typically ranging from 50 to 80 mL/min, was adjusted using the device's menu key. To ensure vascular volume and hemodynamic stability, a balanced salt solution or 9% normal saline was administered. The collection process needed to be completed before heparinization. The obtained APC was stored in citrate-treated bags, maintained at room temperature for a maximum of 6 h before reinfusion to the patient following heparin reversal (Fig. 1). The study's flowchart is depicted in Figs. 2 and 3.

### End points

The primary endpoint of the study focuses on the rate of red blood cell (RBC) transfusions during the perioperative period. Secondary endpoints include the transfusion rates of plasma and platelet, the volume of perioperative transfusions, drainage volume at various intervals (6h, 12 h, 24 h, 48 h, and 72 h), and the incidence of adverse events, including pulmonary insufficiency, infection, stroke, acute kidney injury, liver dysfunction, deep vein thrombosis, pulmonary embolism, re-admission to ICU, major bleeding, re-operation, and 30-day all-cause mortality. The detailed definitions of these endpoints are provided in Supplementary Material 1. Additionally, several other variables are being investigated, such as the duration of mechanical ventilation, ICU length of stay, and overall hospital length of stay.

### Statistical analysis

Data collected from the study were analyzed following the intent-to-treat principle. The normality of continuous variables was assessed through the Shapiro-Wilks test. Continuous variables conforming to a normal distribution were expressed as mean ± standard deviation, whereas those not following a normal distribution were represented by median and interquartile range (IQR). Categorical variables were presented as counts and percentages. The pre-specified analyses of primary and secondary outcomes included all randomized participants. Comparisons between the two groups for continuous variables with a normal distribution were executed via Student's t-test. For continuous variables exhibiting skewed distributions, the Mann–Whitney U test was employed. Categorical variables were compared using the χ^2^ test or Fisher's exact test.

For the primary outcome of this study (RBC transfusion rate) and the key secondary outcomes (fresh frozen plasma [FFP] transfusion rate and platelet transfusion rate), statistical models were constructed to calculate the relative risk (RR) and risk difference (RD) between the APC group and the control group. In Model 1, baseline preoperative characteristics were included as covariates, such as gender, age, body mass index (BMI), medical history, comorbidities, and preoperative ejection fraction (EF). In Model 2, in addition to the above covariates, intraoperative factors were also included as covariates, including the type of surgery, surgical duration, anesthesia time, cardiopulmonary bypass time, cross-clamp time, and intraoperative blood loss. The RR and 95% CI for categorical variables were calculated using a log-binomial model, while the RD and 95% confidence interval (CI) were calculated using the normal approximation method.

Statistical analysis for this study was conducted using Python 3.12.3 (Python Software Foundation, Beaverton, OR, USA) and the Statsmodels library for data analysis and statistical modeling. GraphPad Prism version 8 (La Jolla, CA, USA), and BioRender (https://www.biorender.com/, Toronto, CAN) were used for data visualization. A two-tailed *p*-value < 0.05 was considered statistically significant.

## Results

### Patient Characteristics

From November 2022 to October 2023, a total of 134 patients were enrolled in the study, with 67 patients each allocated to the APC and control groups following the randomization process. All participants completed the trial. The median age of patients in both groups was 49 and 51 years, respectively, with 79.1% of them being male. There were no significant differences observed in baseline characteristics, medical history, or preoperative laboratory test results between the two groups (all *p* > 0.05; Table 1).

**Table 1:** Baseline demographic and clinical characteristics

Variables	APC group (*n* = 67)	Control group (*n* = 67)	t/z/χ^2^	*p* value
Age, y, median (IQR)	49.00 (38.00, 60.00)	51.00 (40.00, 59.00)	−0.519	0.604
Gender male, n (%)	53 (79.10)	53 (79.10)	<.001	>.999
BSA^a^, m^2^, mean (SD)	1.89 ± 0.18	1.85 ± 0.18	1.543	0.125
BMI^†^, kg/m^2^, median (IQR)	25.35 (22.86, 27.47)	24.66 (22.62, 26.83)	−1.081	0.280
LVEF, %, median (IQR)	62.00 (60.00, 66.00)	62.00 (60.00, 65.00)	−0.675	0.500
Medical history, *n* (%)				
Current smoking	12 (17.91)	16 (23.88)	0.722	0.395
ETOH use	5 (7.46)	4 (5.97)	0.119	>.999
Diabetes mellitus	4 (5.97)	2 (2.98)	0.698	0.680
Hypertension	28 (41.79)	33 (49.25)	0.752	0.386
Hyperlipidemia	14 (20.89)	9 (13.43)	1.312	0.360
Previous AF	1 (1.49)	4 (5.97)	1.870	0.365
Previous MI	2 (2.98)	1 (1.49)	0.341	>.999
Previous CVA	4 (5.97)	3 (4.47)	0.151	>.999
Previous cardiac surgery	17 (25.37)	21 (31.34)	0.588	0.443
Preoperative laboratory tests				
Hemoglobin, g/L, mean (SD)	138.21 ± 14.32	140.10 ± 14.67	−0.757	0.451
Leukocyte, 10^9^/L, mean (SD)	6.16 ± 1.46	6.57 ± 1.78	−1.472	0.143
Red blood cell, 10^12^/L, mean (SD)	4.65 ± 0.32	4.70 ± 0.37	−0.825	0.411
PT, s, mean (SD)	13.24 ± 0.33	13.25 ± 0.45	−0.132	0.896
APTT, s, mean (SD)	36.26 ± 3.18	36.11 ± 3.80	0.224	0.807

*APC* Autologous platelet concentrate, *IQR* Inter-quartile range, *BSA* Body surface area, *SD* Standard deviation, *BMI* Body mass index, *LVEF* Left ventricular ejection fraction, *ETOH* Ethyl alcohol, *AF* Atrial fibrillation, *MI* Myocardial infarction, *CVA* Cerebrovascular accident, *PT* Prothrombin time, *APTT* Activated partial thromboplastin time

^a^Calculated as 0.007184 × height^0.725^ × weight^0.425^

^†^Calculated as weight in kilograms divided by height in meters squared

Surgical details are summarized in Table 2. There were no differences in the types of surgery between the two groups. The APC group exhibited a shorter surgical operation time (*p* = 0.003). Moreover, the APC group demonstrated a decrease in intraoperative blood loss (*p* = 0.016), as well as a reduction in the dosage of Factor VII (*p* = 0.040), and an increased usage of calcium gluconate (*p* = 0.042). No significant disparity was observed in the dosage of fibrinogen and prothrombin complex concentrate (PCC) between the two groups.

**Table 2:** Surgical details

Variables	APC group (*n* = 67)	Control group (*n* = 67)	t/z/χ^2^	*p* value
Intravenous crystalloid, ml, mean (SD)	1077.01 ± 522.06	1007.91 ± 615.20	0.701	0.367
Tranexamic acid, *n* (%)	67 (100.00)	67 (100.00)	<.001	>.999
Blood loss, ml, mean (SD)	688.52 ± 166.50	762.58 ± 183.22	−2.449	**0.016**
Calcium gluconate, g, median (IQR)	3.00 (1.00)	2.00 (1.00)	−2.04	**0.042**
Fibrinogen, g, median (IQR)	2.00 (2.00)	2.00 (2.00)	−0.173	0.863
PCC, U, median (IQR)	600.00 (1200.00)	600.00 (1200.00)	−0.319	0.750
Factor VII, mg, median (IQR)	0.00 (0.00)	0.00 (2.00)	−2.054	**0.040**
Operative details				
Aortic arch operation, *n* (%)				
Hemi-arch	6 (8.96)	5 (7.46)	0.099	0.753
Partial-arch	8 (11.94)	10 (14.92)	0.257	0.612
Total-arch	13 (19.40)	22 (32.84)	3.132	0.077
Thoracoabdominal aortic	6 (8.95)	6 (8.95)	0.000	1.000
Aortic root operation, *n* (%)				
None	11 (16.42)	13 (19.40)	0.203	0.652
Repair or plasticity	13 (19.40)	14 (20.90)	0.046	0.829
Bentall	13 (19.40)	14 (20.90)	0.046	0.829
Wheat	0 (0.00)	1 (1.49)	1.008	1.000
David	21 (31.34)	16 (23.88)	0.933	0.334
Concomitant with CABG, *n* (%)	10 (14.92)	15 (22.39)	1.229	0.268
Time duration				
CPB, min, mean (SD)	146.10 ± 45.8	162.41 ± 54.27	−1.794	0.075
Clamp, min, mean (SD)	102.72 ± 34.33	114.80 ± 43.78	−1.696	0.092
Circulatory arrest (*n* = 45)				
* n* (%)	21 (31.34)	24 (35.82)	0.301	0.715
min, mean (SD)	16.14 ± 5.80	17.42 ± 8.04	−0.602	0.551
Anesthesia, min, mean (SD)	56.14 ± 15.12	53.30 ± 19.93	0.906	0.367
Surgical operation, min, mean (SD)	289.93 ± 87.22	340.49 ± 105.98	−3.016	**0.003**
Central venous catheter placement to systemic heparinization, min, mean (SD)				
Root only (*n* = 49)	52.14 ± 7.75	42.15 ± 6.13	4.815	< **.001**
Root + CABG (*n* = 8)	55.50 ± 1.29	48.75 ± 4.65	2.800	0.058
Root + arch (*n* = 42)	56.79 ± 11.42	55.78 ± 10.65	0.295	0.769
Root + arch + CABG (*n* = 17)	56.33 ± 3.67	52.09 ± 4.21	2.072	0.056
Arch only (*n *= 6)	57.67 ± 6.81	51.33 ± 7.57	1.767	0.342
Thoracoabdominal aortic (*n* = 12)	84.50 ± 13.71	81.50 ± 9.89	1.596	0.673

*APC* Autologous platelet concentrate, *SD* Standard deviation, *IQR* Inter-quartile range, *CABG* Coronary artery bypass graft, *CPB* Cardiopulmonary bypass, *PCC* Prothrombin complex concentrate

The *p*-values are indicated in bold if they are statistically significant

We also evaluated the time from central venous catheter placement to systemic heparinization and found that, among patients undergoing isolated aortic root surgery, this interval was significantly longer in the APC group compared to the control group (*p* < 0.001). However, no significant differences were observed between the groups for other types of surgeries.

### Autologous plateletpheresis procedure

67 patients underwent autologous plateletpheresis, which began promptly after central vein catheterization and lasted for an average duration of 42.73 ± 5.54 min. Throughout this procedure, an average of 419.34 ± 99.30 mL of anticoagulant was utilized (Supplementary Table 1). Upon completion, the collected platelets were stored in the transfusion department until the time of protamine reversal, which typically transpired after an approximate interval of 174.72 ± 99.01 min.

### Blood Transfusion

Autologous plateletpheresis can significantly decrease perioperative blood transfusion (Table 3). The APC group exhibited markedly reduced RBC transfusion rates compared to the control group across intraoperative (3% vs 32.8%; *p* < 0.001), postoperative (11.9% vs 28.4%; *p* = 0.018), and total transfusions (14.9% vs 50.7%; *p* < 0.001). This reduction was also reflected in the actual volumes of RBC transfusions. Similar trends were observed in plasma and platelet transfusions.

**Table 3:** Comparison of perioperative blood product utilization between study groups

Variables	APC group (*n* = 67)	Control group (*n* = 67)	t/z/χ^2^	*p* value
Transfusion requirement (quantity if used and percentage of usage)				
Red blood cell (primary outcome)				
Intraoperative, u, median (IQR)	0.00 (0.00, 0.00)	0.00 (0.00, 3.50)	−4.476	<**.001**
* n* (%)	2 (2.99)	22 (32.84)	20.303	<**.001**
Postoperative, u, median (IQR)	0.00 (0.00, 0.00)	0.00 (0.00, 2.00)	−2.548	**0.011**
* n* (%)	8 (11.94)	19 (28.36)	5.612	**0.018**
Total, u, median (IQR)	0.00 (0.00, 0.00)	2.00 (0.00, 4.00)	−4.220	<**.001**
* n* (%)	10 (14.92)	34 (50.75)	19.491	<**.001**
Plasma				
Intraoperative, u, median (IQR)	0.00 (0.00, 0.00)	0.00 (0.00, 0.00)	−2.407	**0.016**
* n* (%)	6 (8.96)	16 (23.88)	5.438	**0.020**
Postoperative, u, median (IQR)	0.00 (0.00, 0.00)	0.00 (0.00, 0.00)	−3.021	**0.003**
* n* (%)	1 (1.49)	11 (16.42)	9.153	**0.002**
Total, u, median (IQR)	0.00 (0.00, 0.00)	0.00 (0.00, 400)	−3.713	<**.001**
* n* (%)	7 (10.45)	25 (37.31)	13.301	<**.001**
Platelet				
Intraoperative, u, median (IQR)	0.00 (0.00, 0.00)	1.00 (1.00, 1.00)	−8.512	<**.001**
* n* (%)	3 (4.48)	52 (77.61)	74.074	<**.001**
Postoperative, u, median (IQR)	0.00 (0.00, 0.00)	0.00 (0.00, 0.00)	−2.426	**0.015**
* n* (%)	1 (1.49)	8 (11.94)	5.836	**0.016**
Total, u, median (IQR)	0.00 (0.00, 0.00)	1.00 (1.00, 1.00)	−9.038	<**.001**
* n* (%)	4 (5.97)	57 (85.07)	84.529	<**.001**

*APC* Autologous platelet concentrate, *IQR* Inter-quartile range

The *p*-values are indicated in bold if they are statistically significant

### Postoperative outcomes

In the APC group, the incidence of major bleeding was significantly lower compared to the control group (1.5% vs 14.9%; *p* = 0.005). Additionally, differences in postoperative drainage volumes were evident between the APC and control groups. Significant differences in postoperative drainage volume were observed between the two groups at various time intervals, specifically at 6 h (230 ml vs 280 ml), 48 h (700 ml vs 790 ml), and 72 h (800 ml vs 930 ml) post-surgery (all *p* < 0.001). The APC group also demonstrated a shorter length of stay in the ICU (50.52 h vs 71.97 h; *p* = 0.043), although no significant difference was found in the total duration of hospital stay between the two groups (Table 4).

**Table 4:** Postoperative outcomes

Variables	APC group (*n* = 67)	Control group (*n* = 67)	t/z/χ^2^	*p* value
Postoperative complications, *n* (%)				
Pulmonary insufficiency	4 (5.97)	5 (7.46)	0.119	>.999
Infection	3 (4.48)	6 (8.96)	1.072	0.300
Stroke	0 (0.00)	2 (2.99	2.030	0.496
Acute kidney injury	1 (1.49)	1 (1.49)	<.001	>.999
Liver dysfunction	3 (4.48)	1 (1.49)	1.031	0.619
Deep vein thrombosis	0 (0.00)	1 (1.49)	1.008	>.999
Re-admission to ICU	2 (2.99)	4 (5.97)	0.698	0.680
Major bleeding	1 (1.49)	10 (14.93)	8.022	**0.005**
Drainage volume, ml, median (IQR)				
6h	230.00 (180.00, 300.00)	280.00 (200.00, 360.00)	−2.521	**0.012**
12h	350.00 (290.00, 470.00)	380.00 (310.00, 510.00)	−1.287	0.198
24h	550.00 (440.00, 680.00)	570.00 (450.00, 730.00)	−1.108	0.268
48h	700.00 (590.00, 890.00)	790.00 (670.00, 1350.00)	−2.010	**0.044**
72h	800.00 (680.00, 1030.00)	930.00 (760.00, 1080.00)	−2.127	**0.033**
ICU length of stay, h, median (IQR)	40.50 (48.00)	47.58 (71.09)	−1.035	**0.301**
Hospital length of stay, d, mean (SD)	14.51 ± 4.28	16.00 ± 9.41	−1.182	0.240

*APC* Autologous platelet concentrate, *ICU* Intensive care unit, *IQR* Inter-quartile range, *SD* Standard deviation

The *p*-values are indicated in bold if they are statistically significant

### Platelet function

At baseline (T0), platelet count and platelet function were comparable between the APC and control groups. A significant reduction in platelet count was observed in the APC group at T1 compared to the control group ([154.81 ± 48.32] × 10^9^/L vs [215.31 ± 49.25] × 10^9^/L; *p* < 0.001). However, no significant differences in platelet count or function were detected between the groups at any subsequent time points (Supplementary Table 2).

### Assessment of Perioperative Blood Transfusion Outcomes

The APC group demonstrated a significant advantage over the control group in reducing the rates of total RBC, FFP, and platelet transfusions during surgery. (for total RBC transfusion rate, Model 1: *p* < 0.001; Model 2: *p* = 0.002; for total FFP transfusion rate, Model 1: *p* = 0.001; Model 2: *p* = 0.034; for total platelet transfusion rate, Model 1: *p* < 0.001; Model 2: *p* < 0.001; Table 5).

**Table 5:** Assessment of perioperative blood transfusion outcomes between the two groups

Outcomes	APC group (*n* = 67)	Control group (*n* = 67)	Risk difference ^a^ (95% CI)	Model 1^b^ (95% CI)	*p* value	Model 2 ^c^ (95% CI)	*p* value
Primary outcome							
RBC transfusion rate							
Intraoperative	2 (2.98)	22 (32.84)	−0.30 (−0.42 ~ −0.18)	0.05 (0.01 ~ 0.29)	0.001	0.08 (0.01 ~ 0.61)	**0.015**
Postoperative	8 (11.94)	19 (28.36)	−0.16 (−0.30 ~ −0.03)	0.30 (0.11 ~ 0.82)	0.018	0.25 (0.06 ~ 1.06)	**0.040**
Total	10 (14.93)	34 (50.75)	−0.36 (−0.51 ~ −0.21)	0.15 (0.06 ~ 0.39)	< .001	0.11 (0.03 ~ 0.45)	**0.002**
Secondary outcomes							
FFP transfusion rate							
Intraoperative	6 (8.96)	16 (23.88)	−0.15 (−0.27 ~ −0.26)	0.42 (0.14 ~ 1.27)	0.124	1.625 (0.307 ~ 8.603)	0.568
Postoperative	1 (1.49)	11 (16.42)	−0.15 (−0.24 ~ −0.06)	0.03 (0.01 ~ 0.40)	0.009	1.073 (0.819 ~ 1.406)	0.067
Total	7 (10.45)	25 (37.31)	−0.27 (−0.41 ~ −0.13)	0.19 (0.07 ~ 0.50)	0.001	0.249 (0.069 ~ 0.900)	**0.034**
PLT transfusion rate							
Intraoperative	3 (4.47)	52 (77.61)	−0.73 (−0.84 ~ −0.62)	0.01 (0.01 ~ 0.05)	< .001	0.01 (0.01 ~ 0.03)	< **.001**
Postoperative	1 (1.49)	8 (11.94)	−0.10 (−0.19 ~ −0.02)	0.03 (0.01 ~ 0.72)	0.031	0.04 (0.01 ~ 0.08)	**0.023**
Total	4 (5/97)	57 (85.07)	−0.79 (−0.89 ~ −0.69)	0.01 (0.00 ~ 0.03)	< .001	0.01 (0.01 ~ 0.03)	< **.001**

*RBC* Red blood cell, *FFP* Fresh frozen plasma, *PLT* Platelet

^a^Using a logistic mixed-effects model to represent the data as risk differences for categorical a outcomes

^b^Preoperative baseline characteristics of the subjects, including gender, age, body mass index, medical history, comorbidities, and preoperative ejection fraction (EF), were included as covariates in the model. The data were represented as relative risks for categorical outcomes using a log-binomial model

^c^In addition to the aforementioned covariates, intraoperative information was also included as covariates in the model, including the type of surgery, surgical duration, anesthesia time, cardiopulmonary bypass time, cross-clamp time, and intraoperative blood loss. The data were represented as relative risks for categorical outcomes using a log-binomial model

The *p*-values are indicated in bold if they are statistically significant

## Discussion

Our study demonstrated that autologous plateletpheresis effectively reduced perioperative blood transfusion requirements, shortened surgical durations, and decreased overall blood product utilization. It also contributed to a lower incidence of major bleeding, reduced postoperative drainage at various intervals, and a shorter length of stay in the ICU. However, these benefits were accompanied by an increased usage of calcium gluconate, which warrants further consideration.

The implementation of PBM strategies during cardiovascular surgeries has shown significant efficacy in reducing allogeneic transfusions, thereby improving clinical outcomes and conserving blood resources [9, 11–14]. However, strategies specifically targeting platelets are relatively limited. The administration of 1 unit of platelets is expected to increase platelet counts by an average of 15–25 × 10^9^/L [15]. Due to concerns regarding immune and viral risks, auto-transfusion has been considered a safer alternative due to reduced interaction with various blood donors [16].

Autologous plateletpheresis aims to sequester the patient's platelet subpopulations from the general circulation, shielding them from exposure to CPB. This sequestration is believed to reduce the risk of platelet dysfunction, maintaining platelet function, expediting hemostasis, and safeguarding tissue microcirculation and endothelial cell integrity [17–19].

This trial introduced innovations based on prior studies. Compared with traditional methods, the modified autologous plateletpheresis maintained an extracorporeal blood volume of about 170 mL, reducing blood dilution and preserving circulatory stability. Additionally, the inclusion criteria for patients in this study were aligned with the standards for voluntary platelet donation set by blood banks, requiring a platelet count 150–450 × 10^9^/L. Patients outside this range, who are typically considered to have a potential risk of bleeding or thrombosis [20, 21], were excluded to ensure the safety of the study.

Although anesthesia, CPB, and aortic clamping durations were similar between groups, the APC group had a shorter surgical duration, less intraoperative bleeding, and reduced Factor VI use, likely reflecting the benefits of APC. Additionally, the APC group required significantly more calcium supplementation, a clinical phenomenon not previously reported in cardiovascular surgery, possibly because most existing studies were retrospective and did not capture this variable. The increased use of calcium supplementation in the experimental group is an expected finding, resulting from citrate anticoagulation during autologous plateletpheresis. Regular monitoring of arterial ionized calcium levels is critical to ensure they remain within the normal range. In cases of clinical signs and documented ionized hypocalcemia, intravenous calcium administration stands as the appropriate treatment [22, 23]. A predefined management strategy was implemented to promptly correct hypocalcemia, which may have contributed to the improved outcomes in the APC group. Future research should pay greater attention to the phenomenon of calcium ion depletion during this process.

The APC group demonstrated significantly lower transfusion rates and volumes of RBCs, platelets, and FFP during the perioperative periods. This aligns with the reduced incidence of major bleeding events in the APC group. Futhermore, postoperative drainage differed significantly between the APC and control groups at various time points. These outcomes suggest that patients in the APC group may experience accelerated post-surgical recovery, potentially leading to shorter ICU stays. However, there was no significant difference in the total length of hospital stay between the two groups, indicating that while patients in the APC group may meet ICU discharge criteria (such as weaning from mechanical ventilation) more quickly, overall discharge criteria may not differ substantially.

Regarding laboratory results, our study found that at T1, platelet counts in the APC group were significantly lower due to the autologous plateletpheresis procedure. However, at T2, although 77.6% of patients in the control group received allogeneic platelets during surgery, only 4.5% of APC group patients required allogeneic platelet transfusion. Despite this, there were no significant differences in platelet counts between the two groups immediately after surgery. It is well-established that platelet hemostatic function is not solely dependent on platelet count, but also on the quality of the thrombus [23]. From T0 to T5, despite the higher use of allogeneic platelets in the control group, no significant differences in platelet function were observed between the two groups. This further supports the clinical effectiveness of autologous plateletpheresis.

Compared to previous studies involving the infusion of aPRP, the results of this study demonstrated more significant clinical effects. This may be due to the lower contamination of RBCs and plasma components in the autologous platelet concentrate prepared by the blood cell separator, as well as a higher platelet count (Note: the quality control standards for machine-collected allogeneic platelets stipulate that the platelet count should be ≥ 2.5 × 10^11^ per bag, while the average platelet count of the final product in this study was 3.07 ± 0.35 × 10^11^ per bag), leading to better transfusion outcomes. In 2012, the National Health Commission of the People's Republic of China issued the *Quality Requirements for Whole Blood and Blood Components *[24], which emphasized minimizing the contamination of RBCs and other blood components during the preparation of aPRP. The free hemoglobin and its degradation products released from ruptured red blood cell membranes in aPRP have cytotoxic effects, causing oxidative stress, nitric oxide depletion, activation of inflammatory pathways, and immune suppression. These factors ultimately lead to microcirculatory dysfunction, local vasoconstriction with vascular injury, and severe tissue damage, triggering the release of macrophage migration inhibitory factors [25]. These cytokines inhibit the migration of monocytes and macrophages, send pro-inflammatory signals to surrounding tissues, suppress stem cell migration, and fibroblast proliferation, thereby causing significant local cellular dysfunction [26]. Therefore, we reasonably hypothesize that the high concentration and high purity of autologous platelets may be one of the potential reasons for the better clinical outcomes observed in the experimental group of this study.

In addition, although autologous plateletpheresis requires central venous catheterization, this procedure does not introduce additional complexity beyond the standard vascular access routinely performed for aortic surgery. While preliminary cost-effectiveness analysis was not feasible due to the trial’s funding structure and the current reimbursement status, future studies incorporating real-world economic evaluations are warranted. Importantly, no adverse events related to the intervention were observed, supporting its preliminary safety. Nonetheless, larger multicenter studies are needed to further validate these findings and assess broader implementation challenges.

This study has several limitations. Firstly, we did not collect allogeneic platelet samples for comparative analysis of platelet count and function, given the standardized process and established safety protocols for blood transfusion. Secondly, the study was confined to three pre-selected clinical wards, aimed at minimizing surgeon-related biases. However, this approach may have inadvertently restricted the trial's statistical power due to the relatively small default sample size. Despite the limitations related to sample size, we observed an overall trend towards improvement in several key clinical parameters, suggesting potential benefits that could be confirmed through larger-scale investigations.

## Conclusions

The improved autologous plateletpheresis technique reduces transfusion requirements, shortens surgical duration, enhances clinical outcomes, and accelerates recovery. However, careful calcium ion monitoring and coordination of pre-CPB preparation times are essential to maintain surgical workflow. Future multicenter studies are needed to further validate these findings.

## Supplementary Information

Supplementary Material 1.

Supplementary Material 2: Table 1.

Supplementary Material 3: Table 2.

## Data Availability

Data will be made available on reasonable request.

## Abbreviations

aPRP: Autologous platelet-rich plasma
APC: Autologous platelet concentrate
BIS: Bispectral index
BMI: Body mass index
CI: Confidence interval
CPB: Cardiopulmonary bypass
EF: Ejection fraction
FFP: Fresh frozen plasma
ICU: Intensive care unit
IQR: Interquartile range
PBM: Patient blood management
PCC: Prothrombin complex concentrate
pRBC: packed red blood cell
PRP: Platelet-rich plasma
RBC: Red blood cell
RD: Risk difference
RR: Relative risk
