# Supplementary material for: Reducing perioperative red blood cell transfusion in adult aortic surgery: innovative application and process optimization of autologous plateletpheresis
Source: Anesthesiol Perioper Sci. 2025 Sep 13;3(3):44. doi: 10.1007/s44254-025-00126-1 (PMC12433371; doi:10.1007/s44254-025-00126-1)
Supplement: Supplementary file 1 — Supplementary Material 1. [file 44254_2025_126_MOESM1_ESM.docx]

**Supplementary Material 1.** The detailed definitions of these endpoints.

| Endpoints | Definitions |
| --- | --- |
| Drainage volume | The volume of fluid collected from pericardial and mediastinal drainage tubes. |
| Pulmonary insufficiency | Diagnosed based on the presence of prolonged ventilator support of more than 24 hours, the development of acute respiratory distress syndrome, pulmonary edema, pneumonia, or reintubation. |
| Infection | Diagnosed based on the presence of clinical signs such as fever, radiographic findings such as new infiltration on chest X-rays or computed tomography scans, positive microbiological culture of an endotracheal aspirate (≥10^6^ colony forming units/mL) or bronchoalveolar lavage (≥10^4^ colony forming units/mL), and leukocytosis. |
| Stroke | Diagnosed based on the presence of a prolonged (72 hours) or permanent neurological deficit (such as paralysis, weakness, or speech difficulty) that is associated with abnormal results on magnetic resonance imaging or computed tomography scans. |
| Acute kidney injury | Diagnosed adheres to the criteria outlined by the Acute Kidney Injury Network (AKIN). For patients without a documented medical history of chronic kidney disease, AKI is diagnosed when there is a rapid decline in renal function occurring within 48 hours, with an absolute increase in serum creatinine of 0.3 mg/dl (equivalent to 26.5umol/L) or a relative increase of 50% or more (exceeding 1.5 times the baseline value), or urine volume less than 0.5 ml/(kg·h) for over 6 hours, with the exclusion of cases involving obstructive nephropathy or dehydration. Serum creatinine at baseline was defined as the serum creatinine value recorded at the time of admission or within one week of clinical presentation. |
| Liver dysfunction | Liver function tests, also known as hepatic function panels, measure the levels of various enzymes, proteins, and other substances in the blood that are produced or processed by the liver. Common markers include:  Alanine Aminotransferase (ALT): Elevated levels can indicate liver damage or disease.  Aspartate Aminotransferase (AST): Elevated AST levels may indicate liver damage but can also be related to heart or muscle issues.  Alkaline Phosphatase (ALP): Elevated ALP may indicate bile duct blockage or liver damage.  Bilirubin: High bilirubin levels can be a sign of liver dysfunction and jaundice.  Furthermore, additional parameters can serve as diagnostic aids:  Prothrombin Time (PT) and International Normalized Ratio (INR): These tests measure blood clotting time and can indicate the liver's synthetic function. An abnormal PT/INR may suggest liver dysfunction.  Albumin Levels: Reduced levels of albumin, a protein produced by the liver, can indicate decreased liver synthetic function.  Imaging Studies: Imaging techniques like ultrasound, CT scans, or MRI can help identify liver abnormalities, including tumors, cirrhosis, or fatty liver.  Biopsy: A liver biopsy involves taking a small sample of liver tissue for microscopic examination. This can provide direct information about liver health and diagnose specific liver conditions.  Clinical Symptoms: Symptoms like jaundice (yellowing of the skin and eyes), abdominal pain, nausea, fatigue, and unexplained weight loss can be indicative of liver dysfunction. |
| Deep vein thrombosis | Clinical Symptoms: swelling, pain, warmth, and redness in the affected limb.  Ultrasound: imaging studies, such as Doppler ultrasound, can detect the presence of a clot in the deep veins. |
| Pulmonary embolism | Clinical Symptoms: shortness of breath, chest pain, rapid heart rate, and in some cases, coughing up blood.  Imaging: a computed tomography pulmonary angiography (CTPA) scan can confirm the presence of a pulmonary embolism. |
| Major bleeding | Bleeding resulting in death, reoperation due to bleeding, intracranial hemorrhage, transfusion of 5 or more units of pRBCs over 48 hours, or pericardial and mediastinal tube drainage exceeding 2000 mL over 24 hours. |
| Re-operation | Excessive chest tube drainage and/or pericardial tamponade requiring re-operation. |
| 30-day all-cause mortality | All-cause mortality within 30 days of the surgical procedure. |

AKI, acute kidney injury; pRBC, packed red blood cell.
