# Supplementary material for: Reducing perioperative red blood cell transfusion in adult aortic surgery: innovative application and process optimization of autologous plateletpheresis
Source: Anesthesiol Perioper Sci. 2025 Sep 13;3(3):44. doi: 10.1007/s44254-025-00126-1 (PMC12433371; doi:10.1007/s44254-025-00126-1)
Supplement: Supplementary file 2 — Supplementary Material 2: Table 1. [file 44254_2025_126_MOESM2_ESM.docx]

Supplementary Table 1. Autologous plateletpheresis details of APC group.

| Variables | APC group  (n=67) |
| --- | --- |
| Autologous platelet count, 10^9^/L, mean (SD) | 1023.00 ± 117.05 |
| Time duration^a^, min, mean (SD) | 42.73±5.54 |
| Dosage of anticoagulant, ml, mean (SD) | 419.34±99.30 |
| Storage time^b^ of APC, min, mean (SD) | 174.72±99.01 |

APC, autologous platelet concentrate; SD, standard deviation.

^a^ represents the total duration from the initiation to the conclusion of platelet collection, excluding the time spent on machine setup, consumables installation, and disassembly.

^b^ represents the time from the end of the platelet collection process to the end of platelet transfusion. This usually occurs after protamine reversal. Until then, platelets were stored according to the requirements of the Transfusion Department.
