# Supplementary material for: Reducing perioperative red blood cell transfusion in adult aortic surgery: innovative application and process optimization of autologous plateletpheresis
Source: Anesthesiol Perioper Sci. 2025 Sep 13;3(3):44. doi: 10.1007/s44254-025-00126-1 (PMC12433371; doi:10.1007/s44254-025-00126-1)
Supplement: Supplementary file 3 — Supplementary Material 3: Table 2. [file 44254_2025_126_MOESM3_ESM.docx]

Supplementary table 2. Perioperative platelet parameters and functional indices between APC and control group.

| Variables | APC group  (n=67) | Control group  (n=67) | *t* | *p* value |
| --- | --- | --- | --- | --- |
| Platelet count (10^9^/L) | | | | |
| T0 | 221.24±56.06 | 219.69±53.09 | 0.165 | 0.807 |
| T1 | 154.81±48.32 | 215.31±49.25 | -7.178 | ＜0.001 |
| T2 | 132.06±34.06 | 129.49±35.24 | 0.430 | 0.668 |
| T3 | 135.91±44.72 | 124.19±38.37 | 1.628 | 0.106 |
| T4 | 139.76±49.51 | 132.11±51.64 | 0.417 | 0.667 |
| T5 | 151.57±61.91 | 131.09±46.54 | 2.165 | 0.032 |
| R (min) | | | | |
| T0 | 6.06±1.07 | 6.16±1.13 | -0.502 | 0.616 |
| T1 | 5.96±1.21 | 6.29±1.28 | -1.537 | 0.127 |
| T2 | 6.28±1.22 | 5.98±1.32 | 1.351 | 0.179 |
| T3 | 5.73±0.87 | 5.43±1.11 | 1.756 | 0.081 |
| T4 | 6.01±1.27 | 6.00±1.02 | 0.015 | 0.988 |
| T5 | 6.47±1.19 | 6.22±0.97 | 1.301 | 0.195 |
| MA (mm) | | | | |
| T0 | 64.55±7.25 | 63.20±7.47 | 1.061 | 0.291 |
| T1 | 60.71±7.64 | 62.58±6.85 | -1.491 | 0.138 |
| T2 | 58.15±9.11 | 58.57±7.13 | -0.297 | 0.767 |
| T3 | 62.59±7.30 | 61.43±6.68 | 0.955 | 0.341 |
| T4 | 66.19±5.71 | 64.53±6.84 | 1.523 | 0.130 |
| T5 | 68.45±5.75 | 67.59±7.15 | 0.768 | 0.444 |
| Angle (°) | | | | |
| T0 | 67.86±5.16 | 66.83±5.54 | 1.103 | 0.272 |
| T1 | 65.47±7.09 | 65.94±6.07 | -0.413 | 0.681 |
| T2 | 64.02±6.61 | 65.09±5.87 | -0.988 | 0.325 |
| T3 | 67.76±4.91 | 66.99±5.29 | 0.870 | 0.386 |
| T4 | 69.76±3.21 | 68.73±5.55 | 1.317 | 0.190 |
| T5 | 69.91±3.93 | 70.39±3.88 | -0.697 | 0.487 |

APC, autologous platelet concentrate; R, reaction time; MA, maximum amplitude; angle, alpha angle.
